# *Sporothrix brasiliensis* Gp70 is a cell wall protein required for adhesion, proper interaction with innate immune cells, and virulence

**DOI:** 10.1016/j.tcsw.2024.100139

**Published:** 2025-01-06

**Authors:** Leonardo Padró-Villegas, Manuela Gómez-Gaviria, Iván Martínez-Duncker, Luz A. López-Ramírez, José A. Martínez-Álvarez, Gustavo A. Niño-Vega, Héctor M. Mora-Montes

**Affiliations:** a Departamento de Biología, División de Ciencias Naturales y Exactas, Campus Guanajuato, Universidad de Guanajuato, Noria Alta s/n, col. Noria Alta, C.P. 36050 Guanajuato, Gto, Mexico; b Laboratorio de Glicobiología Humana y Diagnóstico Molecular, Centro de Investigación en Dinámica Celular, Instituto de Investigación en Ciencias Básicas y Aplicadas, Universidad Autónoma del Estado de Morelos, Cuernavaca, Mor. 62209, Mexico

**Keywords:** Cell wall, Adhesin, Immune recognition, Virulence, *Galleria mellonella*, Phagocytosis, Glycosylation

## Abstract

*Sporothrix brasiliensis* is one of the leading etiological agents of sporotrichosis, a cutaneous and subcutaneous mycosis worldwide distributed. This organism has been recently associated with epidemic outbreaks in Brazil. Despite the medical relevance of this species, little is known about its virulence factors, and most of the information on this subject is extrapolated from *Sporothrix schenckii*. Here, we generated *S. brasiliensis* mutants, where *GP70* was silenced. In *S. schenckii*, this gene encodes a glycoprotein with adhesive properties required for virulence. The *S. brasiliensis GP70* silencing led to an abnormal cellular phenotype, with smaller, round yeast-like cells that aggregate. Cell aggregation was disrupted with glucanase, suggesting this phenotype is linked to changes in the cell wall. The cell wall characterization confirmed changes in the structural polysaccharide β-1,3-glucan, which increased in quantity and exposure at the cell surface. This was accompanied by a reduction in protein content and *N*-linked glycans. Mutant strains with high *GP70*-silencing levels showed minimal levels of 3-carboxy-cis,cis-muconate cyclase activity, this glycoprotein's predicted enzyme function, and decreased ability to bind laminin and fibronectin. These phenotypical changes coincided with abnormal interaction with human peripheral blood mononuclear cells, where production of IL-1β, IL-17, and IL-22 was reduced and the strong dependence on cytokine stimulation *via* mannose receptor was lost. Phagocytosis by monocyte-derived macrophages was increased and virulence attenuated in a *Galleria mellonella* larvae. In conclusion, Gp70 is an abundant cell wall glycoprotein in *S. brasiliensis* that contributes to virulence and proper interaction with innate immnune cells.

## Highlights

• *Sporothrix brasiliensis GP70* encodes a cell wall and secreted protein.
• The *GP70* silencing affected cell morphology, generating smaller, rounded, and aggregated yeast-like cells.
• The *GP70*-silenced mutants showed reduced adhesion to laminin and fibronectin, as well as trace levels of enzyme activity.
• The *GP70* silencing led to defects in the cell wall composition and organization.
• Cells with low Gp70 levels were differentially recognized by human mononuclear cells and monocyte-derived macrophages.
• The *GP70*-silenced mutants showed attenuation in virulence when interacting with *Galleria mellonella* larvae.

## 1 Introduction

The *Sporothrix* genus groups filamentous fungi, most of them with a saprophytic lifestyle, and are found in different environmental niches (de Beer et al., 2016; de Beer et al., 2022). The *Sporothrix* molecular analyses helped to subclassify them into clades, and the pathogenic clade includes *Sporothrix schenckii, Sporothrix brasiliensis, Sporothrix globosa,* and *Sporothrix luriei* (de Beer et al., 2016). These are the most frequent etiological agents of sporotrichosis, a fungal infection of the skin and subcutaneous tissues of human beings and other mammals (Bonifaz and Vázquez-González, 2013). Sporotrichosis cases have been reported from different continents, being considered a worldwide distributed infection. However, epidemic outbreaks have been reported in Peru, Mexico, China, Japan, India, South Africa, Australia, Colombia, and Venezuela (Chakrabarti et al., 2015, Gómez-Gaviria et al., 2023a, Gómez-Gaviria et al., 2023b). It is noteworthy to mention the outbreak in Brazil caused by *S. brasiliensis*, which has affected thousands of humans and animals (Gremião, 2021, Gómez-Gaviria et al., 2023a, Gómez-Gaviria et al., 2023b, Xavier et al., 2023). The infection has not respected political borders, and *S. brasiliensis*-caused infections have been reported in Argentina, Paraguay, Panama, the United Kingdom, and Chile (Gómez-Gaviria et al., 2023a, Gómez-Gaviria et al., 2023b, Xavier et al., 2023). Sporotrichosis is a life-threatening infection in immunocompromised patients, where a disseminated form may affect deep-seated organs (Lopez-Romero, 2011). However, most of the sporotrichosis cases are benign lymphocutaneous, and fixed cutaneous infections associated with low mortality rates (Vásquez-del-Mercado et al., 2012; Bonifaz and Vázquez-González, 2013).

The epidemiology, diagnosis, and treatment of infection caused by *S. brasiliensis* have been extensively investigated in recent years (Etchecopaz et al., 2021; Rodrigues et al., 2022; Xavier et al., 2023; Poester et al., 2024; Santos et al., 2024). The study of animal sporotrichosis, in particular the feline disease, has changed the paradigm of the infection route, which was considered only by traumatic contact with plant debris where the fungus grows (Lopes-Bezerra et al., 2018a, Lopes-Bezerra et al., 2018b). Nowadays, the zoonotic route of infection for *S. brasiliensis* and other *Sporotrhix* species is also recognized (Santos et al., 2024), and in the case of *S. brasiliensis*, the infection by contact with yeast-like cells from cats' open skin lesions has been documented (Rodrigues et al., 2013). Despite this significant advance in the knowledge of this fungal species, its basic biological traits remain poorly studied (Gómez-Gaviria et al., 2023a, Gómez-Gaviria et al., 2023b). One naïve mistake in biology is assuming that the biology of a well-studied species may be like others, particularly close phylogenetic relatives. In this case, *S. schenckii* is the most studied species of the *Sporothrix* genus (Mora-Montes et al., 2015), and researchers may wrongly assume *S. brasiliensis* biology is similar to *S. schenckii*.

Adhesins, hydrolytic enzymes, dimorphism, resistance to oxidative and nitrosative stress, thermotolerance, pigment production, and immune evasion are among the canonical virulence factors found in medically relevant fungal species (Staniszewska, 2020; Tamez-Castrellón et al., 2020). However, the study of *S. brasiliensis* virulence factors is scarce (Gómez-Gaviria et al., 2023a, Gómez-Gaviria et al., 2023b), despite this species being considered the most virulent of the *Sporothrix* genus (Arrillaga-Moncrieff et al., 2009; Castro et al., 2013). Adhesion is among the first steps during the host-fungus interaction, as fungal cells can adhere to host cells and extracellular components (Teixeira et al., 2009). *Sporothrix* cells can adhere to fibronectin, fibrinogen, laminin, and type II collagen (Lima et al., 1999; Lima et al., 2001; García-Carnero et al., 2021; López-Ramírez et al., 2024), and epithelial cells (Sandoval-Bernal et al., 2011). Adhesins are usually cell wall proteins that are transported to the extracellular compartment by the secretory pathway or moonlighting proteins localized at the cell wall by non-conventional routes (Cota and Hoyer, 2015; Arvizu-Rubio et al., 2022).

Thus far, Gp70 is the most characterized adhesin in *S. schenckii* and has also been found in *S. globosa* and *S. brasiliensis* (Castro et al., 2013; Rodrigues et al., 2015). This is a cell wall glycoprotein that binds fibronectin and laminin *in vitro*, and to the mouse tail dermis *in vivo* (Ruiz-Baca et al., 2009; Teixeira et al., 2009; López-Ramírez et al., 2024). This is likely a moonlighting protein, as the peptide also has 3-carboxy-*cis*,*cis*-muconate cyclase activity (Rodrigues et al., 2015; Martínez-Álvarez et al., 2019; López-Ramírez et al., 2024). In *S. brasiliensis*, *GP70* encodes a 43-kDa polypeptide that is extensively glycosylated, generating glycoforms with molecular weights ranging from 60 to 70 kDa (Rodrigues et al., 2015). The 60-kDa glycoforms are more abundant in the *S.brasiliensis* cell wall than in *S. schenckii* (Ruiz-Baca et al., 2011; Ruiz-Baca et al., 2014; Rodrigues et al., 2015). In *S. schenckii*, *GP70* silencing led to changes in the cell wall composition, reduced adhesion to laminin and fibrinogen, and virulence attenuation (López-Ramírez et al., 2024). However, the role of this glycoprotein in the *S. brasiliensis*-host interaction may be different, as low Gp70 levels were associated with high virulence in *S. brasiliensis* (Castro et al., 2013). Contrary to this observation, monoclonal anti-Gp70 antibodies were capable of controlling experimental sporotrichosis caused by *S. brasiliensis*, and Gp70 peptides were found as good candidates to develop a CD4^+^-dependent protective immune response against subcutaneous sporotrichosis (de Almeida et al., 2015; de Almeida et al., 2017; de Almeida et al., 2018).

Here, to assess the biological role of Gp70 in the *S. brasiliensis*-host interaction, silenced mutants were generated and the phenotype was analyzed. In particular, cell wall composition, adhesion to extracellular matrix components, interaction with immune cells, and virulence in the alternative model *Galleria mellonella* were investigated.

## 2 Material and methods

### 2.1 Strains and culture media

The strains used and generated here are listed in Table 1. Mycelia were propagated at 28 °C for 7 days in YPD medium, pH 4.5 (1 % [*w*/*v*] yeast extract, 2 % [w/v] gelatin peptone, 3 % [w/v] dextrose, and 2 % [w/v] agarose). Conidia were harvested from solid cultures and used either for mycelia propagation in YPD broth, pH 4.5, or induction of cell dimorphism into yeast-like cells. For the latter, conidia were incubated in YPD broth, pH 7.8 for 4 days at 37 °C and 120 rpm, as described (Martínez-Álvarez et al., 2017). Yeast-like cells were washed three times with deionized water and kept at −20 °C until used for phenotypical characterization. When required, cells were heat inactivated by incubating at 60 °C for 2 h, as described (Martínez-Álvarez et al., 2017). To disrupt cell aggregates, yeast-like cells were incubated with 3 U β-glucanase from *Trichoderma longibrachiatum* for 1 h at 37 °C (Bauer et al., 1973; Kanda et al., 1976). *Agrobacterium tumefaciens* AGL-1 was grown in LB medium (0.5 [w/v] yeast extract, 1 % [w/v] gelatin peptone, and 1 % [w/v] NaCl) at 28 °C. For *Agrobacterium*-mediated transformation, fungal cells were selected on YPD plates containing 150 μg mL^−1^ hygromycin B (GoldBio, St Louis, MO, USA), pH 4.5 (Lozoya-Pérez et al., 2019).

**Table 1:** *Sporothrix schenckii* strains used in this study.

Strain	Genotype	*GP70* expression (%)^a^	Insertional events of pBGgHg^b^	Reference
5110 ATCC MYA 4823	Wild-type	100.0 ± 3.2	N.D.	(Castro et al., 2013)
HSB1	5110 ATCC MYA 4823 transformed with pBGgHg	99.2 ± 2.3	1.2 ± 0.6	This work
HSB2	5110 ATCC MYA 4823 transformed with pBGgHg	98.9 ± 3.0	1.4 ± 0.3	This work
HSB3	5110 ATCC MYA 4823 transformed with pBGgHg-GP70	44.8 ± 1.2	3.1 ± 0.7	This work
HSB4	5110 ATCC MYA 4823 transformed with pBGgHg-GP70	47.3 ± 2.2	3.2 ± 0.3	This work
HSB5	5110 ATCC MYA 4823 transformed with pBGgHg-GP70	49.5 ± 2.7	3.3 ± 0.5	This work
HSB6	5110 ATCC MYA 4823 transformed with pBGgHg-GP70	9.2 ± 1.1	3.1 ± 0.3	This work
HSB7	5110 ATCC MYA 4823 transformed with pBGgHg-GP70	7.7 ± 2.1	3.2 ± 0.4	This work
HSB8	5110 ATCC MYA 4823 transformed with pBGgHg-GP70	6.5 ± 1.5	3.3 ± 0.5	This work

a Quantified by RT-qPCR. ^b^ Estimated by qPCR. In both cases, data were normalized using *L6* as a reference gene. Data are means ± SD of three independent experiments performed in duplicates.

### 2.2 Gene silencing

The binary plasmid pBGgHg-GP70 used previously for *GP70* silencing in *S. schenckii* was used to transform *S. brasiliensis* cells (Lozoya-Pérez et al., 2018; López-Ramírez et al., 2024). This is a pBGgHg-based plasmid (Chen et al., 2000), which contains a 295 bp-fragment of the 5′ end of the *GP70* open reading frame. This fragment is 98.3 % identical in both species, containing only five nucleotide changes scattered across this gene fragment. *A. tumefaciens* AGL-1 cells transformed with the binary plasmid and previously activated with 200 μM acetosyringone (Sigma-Aldrich, San Luis, MO, USA) (Lozoya-Pérez et al., 2018) were mixed with 1 × 10^5^ conidia and co-incubated for 72 h at 28 °C on a cellophane disk on top of YPD, pH 4.5 agar. Then, the cellophane was changed to plates containing fresh medium supplemented with 150 μg mL^−1^ hygromycin B and 200 μM cefotaxime (Sigma-Aldrich), and incubated for 72 h at 28 °C. The selected transformant cells underwent five monoconidial passages in YPD, pH 4.5 and 150 μg mL^−1^ hygromycin B; and were subjected to three dimorphism events in YPD, pH 7.8 and 150 μg mL^−1^ hygromycin B to remove non-transformed nuclei (Lozoya-Pérez et al., 2018).

### 2.3 Gene expression analysis and quantification of binary plasmid insertional events

Gene expression was analyzed by RT-qPCR in a thermocycler StepOne Plus (Life Technologies, Carlsbad, CA, USA), using the StepOne software V 2.2 (Life Technologies) and the 2^-∆∆Ct^ method (Livak and Schmittgen, 2001). For this purpose, total RNA was extracted from 4-day-grown yeast-like cells as described (Robledo-Ortiz et al., 2012), cDNA was synthesized and purified using an in-house chromatography-based protocol (Trujillo-Esquivel et al., 2016; Trujillo-Esquivel et al., 2017), and quantified in a NanoDrop 2000 (Thermo Fisher Scientific, Waltham, MA, USA). The amplification reactions contained SYBR Green PCR Master Mix (Life Technologies) and the primers 5’-TTCCTGAGCAGCCTGGACGG and 5’-AGGTCTCCGTCAGGTTGGG, which amplify the 295-bp sense/antisense *GP70* region cloned into the pBGgHg-GP70 binary vector (López-Ramírez et al., 2024). The gene encoding the ribosomal protein L6 was used for data normalization, as this was previously reported to be a constitutive expression gene in *S. schenckii* (Trujillo-Esquivel et al., 2017). This was amplified with the primers 5’-ATTGCGACATCAGAGAAGG and 5’-TCGACCTTCTTGATGTTGG. The reference condition in these reactions was the parental strain. For analysis of the putative ortholog of *S. schenckii MKC1* (accession code at GenBank XM_040760024) the following primer pair was used 5’-ACCAGCTCAACCAGATCCTC and 5’-CAAAGTTGAAGGTGGTGGGG. A similar strategy was used to estimate the number of insertional events of the binary vector within the fungal genome, using genomic DNA and qPCR.

### 2.4 Enzyme assays

The 3-carboxy-cis,cis-muconate cyclase activity was measured as described (Martínez-Álvarez et al., 2019). Four-day-grown yeast-like cells were pelleted, washed twice with citrate buffer, pH 6.8, and disrupted in an MSK cell homogenizer (Braun, Melsungen, Germany). The homogenate was centrifuged at 3000 x*g* for 15 min at 4 °C, the supernatant was saved and used to determine 3-carboxy-cis,cis-muconate cyclase activity. Aliquots containing 50 μg protein were used to read the absorbance at 260 nm and then 10 mM 3-carboxy-cis-cis-muconate was included. Reactions were incubated for 5 min at 30 °C. The absorbance at 260 nm was read again. Production of 3-carboxymuconolactone was estimated by the change in the absorbance at 260 nm, which was associated with the generation of the product (Thatcher and Cain, 1975). Alternatively, aliquots containing 1.0 × 10^7^ yeast-like cells were suspended in 20 mL of YPD, pH 7.8 for 4 days, cells pelleted, the supernatant saved, diallized against citrate buffer, pH 6.8, and proteins concentrated in an Amicon Ultra centrifugal filter with Ultracel-3 K (Sigma-Aldrich) and kept at −20 °C until used. This protein preparation was used to assess enzyme activity as well. In both cases, the specific activity was defined as Δ_260nm_ min^−1^ mg protein^−1^.

### 2.5 Evaluation of cell adhesion

We followed a standardized methodology, similar to an ELISA (Lima et al., 1999). The solid phase was Nunc MaxiSorp™ flat-bottom 96-well microplates, which were coated for 3 h at room temperature with 0.05 % (*w*/*v*) PBS-Tween 20 and 1 μg of the following extracellular matrix components: bovine type II collagen, human laminin, human elastin, human fibrinogen, human recombinant fibronectin, human recombinant thrombospondin-1, or human type-I collagen (all from Sigma-Aldrich). Plates were blocked with 1 % (w/v) bovine serum albumin (BSA) in PBS and incubated overnight at 4 °C. Then, 5 × 10^6^ yeast-like cells in 100 μL PBS were added to wells, incubated for 1 h at 37 °C, 100 μL of rabbit polyclonal anti-rHsp60 diluted at 1:3000 were added (García-Carnero et al., 2021), plates incubated 2 h at room temperature, 100 μL of goat anti-rabbit IgG-peroxidase antibody diluted 1:5000 (Sigma-Aldrich) was added, and further incubated for 2 h at room temperature. Then, 0.1 mg mL^−1^ 2,2′-azino-bis(3-ethylbenzothiazoline-6-sulfonic acid) diammonium salt and 0.006 % (*v*/v) hydrogen peroxide were added to wells and incubated at room temperature for 20 min. Finally, color development was stopped with 2 N sulfuric acid, and absorbance was read at 450 nm in a Varioskan LUX Multimode Microplate Reader (Thermo Fisher Scientific). During the whole process, plates were washed three times with 0.05 % (w/v) PBS-Tween 20 in between steps. When mentioned, yeast-like cells were incubated with preimmune sera or polyclonal anti-rGp70 antibodies diluted at 1:3000 (Martínez-Álvarez et al., 2019) for 60 min at 37 °C, before inclusion in the ELISA method. Mock interactions, referred to here as controls, are wells where yeast-like cells were not included, and substituted with PBS.

### 2.6 Cel wall and protein glycosylation analyses

Cells grown for 4 days at 37 °C in YPD, pH 7.8 were disrupted in an MSK cell homogenizer (Braun, Melsungen, Germany), as reported (Mora-Montes et al., 2010). Cell walls were cleansed by serial incubations with hot SDS, β-mercaptoethanol, and NaCl, before being hydrolyzed with 2 M trifluoroacetic acid (Sigma-Aldrich), as reported (Mora-Montes et al., 2007). The digestions were neutralized with 1 N NaOH and samples were used for analysis in a Dionex system (Thermo Fisher Scientific), where High-Performance Anion-Exchange Chromatography with Pulsed Amperometric Detection (HPAEC-PAD) was performed. Sugar separation was conducted in a CarboPac PA-1 column with a pre-guard CarboPac PA-1 column, with 3.5 % (w/v) 200 mM NaOH and a flux rate of 1 mL min^−1^. Data were normalized to the monosaccharide content quantified in one mg of dry cell wall, using the phenol‑sulfuric acid method (Dubois et al., 1956).

The quantification of cell wall proteins was performed in alkali-hydrolyzed walls, using the Pierce BCA Protein Assay (Thermo Fisher Scientific), as reported (Mora-Montes et al., 2007).

To assess changes in the distribution of chitin and β-1,3-glucan content within the cell wall, chitin was labeled by incubating yeast-like cells with 500 μg mL^−1^ fluorescein isothiocyanate conjugated-wheat germ agglutinin (WGA-FITC; Sigma-Aldrich) for 60 min at room temperature, as described (Mora-Montes et al., 2011). For β-1,3-glucan detection, cells were incubated with 5 μg mL^−1^ IgG Fc-Dectin-1 chimera (Graham et al., 2006) for 40 min at room temperature, and then with 1 μg mL^−1^ donkey anti-Fc IgG-FITC (Sigma-Aldrich) for 40 min at room temperature (Marakalala et al., 2013). In both cases, polysaccharide labeling was examined under fluorescence microscopy in a Zeiss Axioscope-40 microscope (Carl Zeiss AG, Jena, Germany) and an Axiocam MRc camera (Carl Zeiss AG). Three hundred cells per biological replicate were analyzed per strain or condition. The cell-associated fluorescence was acquired with Adobe Photoshop™ CS6, as reported (Perez-Garcia et al., 2016). The fluorescence associated with HK cells, where all the polysaccharides are accessible to lectins (Martínez-Álvarez et al., 2017), was regarded as 100 % and used for data normalization.

To assess the content of cell wall *O*-linked or *N*-linked glycans, 1 × 10^9^ yeast-like cells were β-eliminated or incubated for 20 h at 37 °C with 25 U endoglycosidase H (New England Biolabs), respectively (Lozoya-Pérez et al., 2019; García-Carnero et al., 2023). In both cases, cells were pellet by centrifuging, and glycans were saved from the supernatants, which were used for total sugar content quantification with the phenol‑sulfuric acid method (Dubois et al., 1956). In addition, glycans were acid hydrolyzed and monosaccharides were separated by HPAEC-PAD, as reported. (Mora-Montes et al., 2012).

### 2.7 Ethics statement

This study was conducted following the Declaration of Helsinki. The Institutional Research Ethics Committee (CEPIUG) approved the use of primary human cells in this study (Ref. CEPIUG-P13–2023). Only healthy adult volunteers who signed the informed consent were enrolled.

### 2.8 Interaction with human peripheral blood mononuclear cells

Human venous blood anticoagulated with EDTA was used to isolate peripheral blood mononuclear cells (PBMCs) by differential centrifugation in Histopaque-1077 (Sigma-Aldrich), as reported (Endres et al., 1988). Round-bottom 96-well microplates were used to perform cell-cell interactions as follows: each well contained 100 μL 1 × 10^5^ yeast-like cells and 100 μL 5 × 10^5^ PBMCs both in RPMI 1640 Dutch modification (supplemented with 2 mM glutamine, 0.1 mM pyruvate and 0.05 mg mL^−1^ gentamycin; all from Sigma-Aldrich). The cellular interactions were incubated at 37 °C for 24 h with 5 % (*v*/v) CO_2_, then plates were centrifuged for 10 min at 3000 x*g* at 4 °C, the supernatants saved, and stored at −20 °C. For interleukin 17 (IL-17) and interleukin 22 (IL-22) stimulation, human PBMCs were added with 10 % (v/v) human pooled serum, and the yeast-like cells were previously killed with UV light (four doses of UV radiation at 100 mJ cm^2–1^ in a UV-DNA crosslinker CL-3000 from Analytik Jena, Upland, CA, USA). The microplates were incubated at 37 °C for 7 days with 5 % (*v*/v) CO_2_ (Tóth et al., 2013) and supernatant saved and stored at −20 °C. The interleukin-1β (IL-1β), interleukin-6 (IL-6), interleukin-10 (IL-10), interleukin-17 (IL-17), interleukin-22 (IL-22), and tumor necrosis factor-alpha (TNFα) were quantified from supernatants with DuoSet ELISA Development kits (R&D Systems, Minneapolis, MN, USA). In all cases, wells containing only human PBMCs were included as controls, and the measurements were subtracted in all experimental wells.

When indicated, the PBMCs were pre-incubated for 1 h at 37 °C and 5 % (v/v) CO_2_ with any of the following antagonistic reagents: 200 μg mL^−1^ laminarin (Sigma-Aldrich), 10 μg mL^−1^ of anti-mannose receptor (MR) (Thermo-Fisher Scientific, MA5–44033), or 10 μg mL^−1^ anti-TLR4 antibody (Santa Cruz Biotechnology, Dallas, TX, USA sc-293,072). Preincubations with Isotype matched, 10 μg mL^−1^ irrelevant IgG1 antibody (Santa Cruz Biotechnology, Cat. No. sc-52,003) were used as control. Even though the antibodies were negative for bacterial lipopolysaccharide, assessed with a *Limulus* amebocyte lysate (Sigma-Aldrich), interactions involving preincubation with antibodies were performed in the presence of 5 μg mL^−1^ polymyxin B (Sigma-Aldrich) as described (Martínez-Álvarez et al., 2017).

### 2.9 Analysis of phagocytosis by human monocyte-derived macrophages

Human PBMCs were stimulated to undergo differentiation to macrophages by incubating with recombinant human granulocyte-macrophage colony-stimulating factor (Sigma-Aldrich), as described (Perez-Garcia et al., 2016). *S. brasiliensis* yeast-like cells uptake by human monocyte-derived macrophages was analyzed by flow cytometry in a FACSCanto II equipped with a FACSDiva acquisition system (Becton Dickinson, Franklin Lakes, NJ, USA), as described (González-Hernández et al., 2017, Gómez-Gaviria et al., 2023a, Gómez-Gaviria et al., 2023b). Yeast-like cells were labeled with 1 mg mL^−1^ Acridine Orange (Sigma-Aldrich) as described (Abrams et al., 1983). A total of 50,000 events were collected per biological replicate, gating for immune cells, previously stained with 1.25 mg mL^−1^ Trypan Blue (Hernández-Chávez et al., 2018). Fluorescent signals were obtained from the FL1 (green) and FL2 (red) channels. Trypan blue quenches Acridine Orange, generating a green fluorescence, and these cells were classified at the early stage of phagocytosis. Acridine Orange in an acidic environment, such as the mature phagolysosome emits a red fluorescent signal, and therefore cells positive for the red channel were considered in the late stage of the phagocytosis (Abrams et al., 1983) Cells positive for both the green and red channels were grouped in the intermediate stage of phagocytosis (Hernández-Chávez et al., 2018; Lozoya-Pérez et al., 2019). When required, human monocyte-derived macrophages were preincubated with 200 μg mL^−1^ laminarin (Sigma-Aldrich) for 1 h at 37 °C and 5 % (v/v) CO_2._

### 2.10 Virulence assays

The analysis of virulence was performed in *Galleria mellonella* larvae as previously described and using an in-house colony already established (Clavijo-Giraldo et al., 2016). Larvae were kept on a diet based on corn bran and honey (García-Carnero et al., 2020), and only healthy animals were included in this study. This condition was defined as those larvae with a length of 1.2–1.5 cm, absence of body melanization, active behavior, and irritability to physical contact (Clavijo-Giraldo et al., 2016; García-Carnero et al., 2020). Fungal inocula were prepared at a concentration of 1 × 10^7^ yeast-like cells mL^−1^ and from these 10 μL (1 × 10^5^ cells) were injected in the last left pro-leg, with a Hamilton syringe and a 26-gauge needle (Clavijo-Giraldo et al., 2016). Inoculated larvae were kept in Petri dishes at 37 °C and survival was monitored daily for two weeks. Animal death was defined as body melanization throughout the whole larva body and lack of response to physical contact. Each fungal strain was inoculated into groups containing 30 larvae. A mock group, inoculated with PBS was included as control. Upon animal death or at the end of day 15, for animals alive at the end of the experiment, hemolymph was anticoagulated, collected by larva decapitation, and used to quantify colony-forming units (CFUs), as reported (García-Carnero et al., 2020). For hemocyte counting, cytotoxicity, and phenol oxidase activity, groups containing 10 larvae were inoculated as described, incubated at 37 °C and 24 h post-inoculation larva were decapitated, hemolymph collected, anticoagulated, and used for the analysis of the mentioned parameters (Lozoya-Pérez et al., 2020). Cytotoxicity (defined here are the release of lactate dehydrogenase, LDH, to the extracellular compartment) and phenoloxidase activity were measured in cell-free hemolymph, using the Pierce LDH Cytotoxicity Assay (Thermo Fisher Scientific), and 20 mM 3,4-dihydroxyDL-phenylalanine (Sigma-Aldrich), respectively (García-Carnero et al., 2020).

### 2.11 Statistical analysis

Statistical analysis was performed using GraphPad Prism 6 software. Data were first analyzed with Dunnett's test. When the *P* value was <0.05, further statistical analysis was performed. Data normality was analyzed with the Shapiro-Wilk test. Cytokine stimulation, phagocytosis by human cells, and parameters obtained from the *Galleria*-fungus interaction did not show normality, and the Mann-Whitney *U* test was used for further analysis. Experiments with human cells were performed in duplicate with samples from eight healthy donors. Animal survival experiments were analyzed with the Log-rank test and are reported in Kaplan-Meier survival curves. Other experiments were performed at least three times in duplicate. Since results showed normality, these were analyzed with the unpaired *t*-test. In all cases, the significance level was set at *P* < 0.05.

## 3 Results

### 3.1 *GP70* silencing in *Sporothrix brasiliensis*

**Supplementary Fig. S1:**
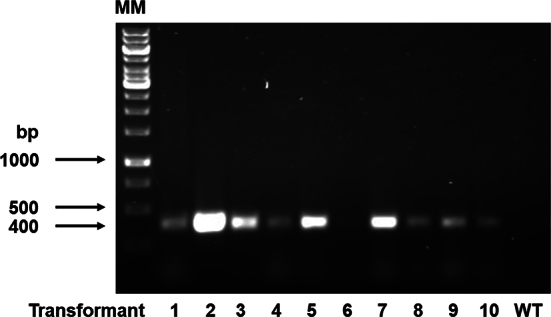
**PCR amplification of a fragment of pBGgHg-GP70.** Upon monoconidial passes and induction of dimorphism, genomic DNA was isolated from transformants and used to amplify a fragment of the gene conferring resistance to hygromycin B, located within pBGgHg-GP70. The primer pair used was 5´-GAAGAATCTCGTGCTTTCAG and 5´-CACAGTTTGCCAGTGATACA, which amplified a 400 bp amplicon. MM, molecular markers; WT, strain 5110 ATCC MYA 4823. Transformants 1,2,3,5, 7, and 8 are referred to in the text as HSB3, HSB4, HSB5, HSB6, HSB7, and HSB8, respectively.

We followed the reported methodology for gene silencing in *S. schenckii* (Lozoya-Pérez et al., 2018), as *S. schenckii* and *S. brasiliensis* share several phenotypical and genomic traits that help us to hypothesize this methodology is suitable also for this species (Teixeira et al., 2014, Lopes-Bezerra et al., 2018a, Lopes-Bezerra et al., 2018b). Upon *A. tumefasciens*-mediated transformation, we obtained 331 transformants that were selected with hygromycin B and by monoconidial passages. Of this 206 transformants were selected and PCR confirmed the presence of pBGgHg-GP70 (see supplementary Fig. 1S). These strains were used to analyze *GP70* expression by RT-qPCR, and from them, six colonies were selected for detailed phenotypical characterization, named HSB3-HSB8 (Table 1). Strains HSB3, HSB4, and HSB5 showed an intermediate level of *GP70* silencing, whilst strains HSB6, HSB7, and HSB8 showed minimal levels of *GP70* expression (Table 1). To assess the contribution of the binary vector to the phenotype of mutant strains, the wild-type (WT) strain 5110 ATCC MYA 4823 was also transformed with the empty pBGgHg vector. Following the already described selection strategy, two random strains (HSB1 and HSB2) were selected and used as control strains. Both strains showed similar *GP70* expression levels as the WT strain (Table 1).

**Fig. 1:**
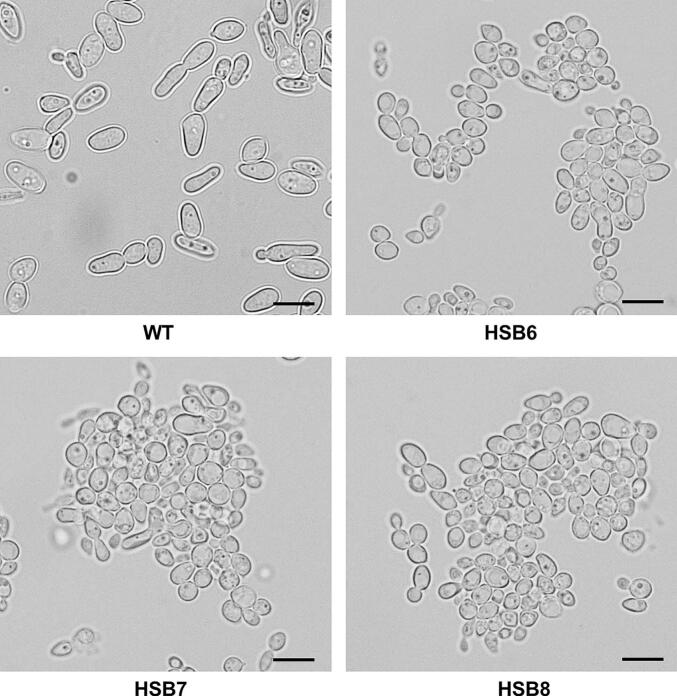
***Sporothrix brasiliensis* yeast-like cell morphology*.*** Representative light microscopy images of *S. brasiliensis* wild-type (WT) strain, and three mutant strains with high levels of *GP70* silencing (HSB6, HSB7, and HSB8). WT, strain 5110 ATCC MYA 4823.Scale bars = 5.0 μm.

**Fig. 2:**
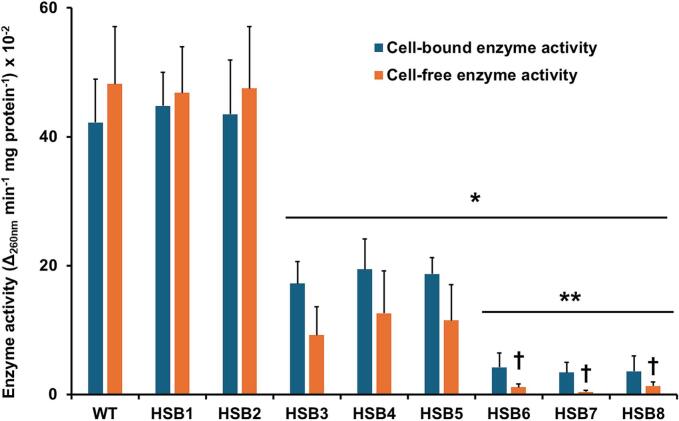
**Analysis of 3-carboxy-cis,cis-muconate cyclase activity in *Sporothrix brasiliensis* wild-type, control, and *GP70*-silenced strains.** Cell homogenates prepared from yeast-like cells (cell-bound enzyme activity) were used to measure enzyme activity using 3-carboxy-cis-cis-muconate as substrate. Alternatively, the dialyzed and concentrated media where yeast-like cells were grown (cell-free enzyme activity) was used in enzyme assays. Data are means ± SD of three biological replicates performed in duplicates. The Dunnett's test and then unpaired *t*-test were used for data analysis. *P < 0.05 when compared to WT or control cells. **P < 0.05 when compared to strains HSB3-HSB5. ^†^*P* < 0.05 when compared to cell-bound enzyme activity. WT, strain 5110 ATCC MYA 4823. Strains HSB1 and HSB2 were transformed with pBGgHg; while HSB3-HSB8 with pBGgHg-Gp70.

The strategy used does not control the binary vector integration site, but we can select strains with only one insertional event within the fungal genome. This was assessed by qPCR, amplifying the *GP70* fragment used to generate the sense and antisense regions of the binary plasmid. Consequently, one copy is expected to be amplified from the WT strain, while three copies of this region would be amplified from a mutant strain with one copy of the binary plasmid integrated outside of the *GP70* locus. The control strains transformed with the empty vector showed only one copy of the *GP70* region, while the six silenced strains showed three copies of it, indicating all the silenced strains contained one insertional event within the genome (Table 1). The WT, control strains, and *GP70*-silenced mutants showed similar doubling times in both morphologies, hypha and yeast-like cells (hypha 3.8 ± 0.8 h and yeast-like cells 8.1 ± 0.7 h, respectively). Colony morphology did not show any significant change. In terms of cell morphology, the WT, control, and mutant with intermediate *GP70* silencing levels (HSB3-HSB5) showed the conventional cigar-shape morphology (Fig. 1, and Fig. 2S), but the highly *GP70* silenced strains (HSB6-HSB8) showed a rounded form that was smaller and formed cell aggregates (Fig. 1). These were disrupted when cells were treated with glucanase. Thus, the phenotypical characterization was performed with glucanase-treated cells, including those strains that did not form aggregates.

The *S. schenckii* Gp70 has an amino acid sequence similar to 3-carboxy-cis,cis-muconate cyclases found in other fungal species (Rodrigues et al., 2015), and its enzyme activity has been experimentally confirmed (Martínez-Álvarez et al., 2019; López-Ramírez et al., 2024). Since the *S. brasiliensis* Gp70 is 95.16 % identical to the *S. schenckii* glycoprotein (Rodrigues et al., 2015), it is feasible to hypothesize this glycoprotein has enzyme activity. Results showed that Gp70 activity was similar in the WT and control strains, and this was partially reduced in strains HSB3, HSB4, and HSB5, and barely detected in strains HSB6, HSB7, and HSB8, confirming that the enzyme activity was associated with the *GP70* product and its transcriptional silencing (Fig. 2). It was previously reported that Gp70 may be found in the extracellular compartment of *S. brasiliensis* yeast-like cells, being a major cell-free antigen for this species (Almeida-Paes et al., 2012; Rodrigues et al., 2015). Thus, we also assayed the 3-carboxy-cis,cis-muconate cyclase activity in the cell-free extract from the medium where *S. brasiliensis* yeast-like cells were grown. The enzyme activity in these preparations was similar to that measured in cell homogenates from WT and control strains (Fig. 2). Similar to the cell-associated enzyme activity, this was partially loss in the strains with intermediate silencing levels (HSB3, HSB4, and HSB5), and significantly reduced in the highly silenced strains (HSB6, HSB7, and HSB8; Fig. 2). Collectively, these data indicate that silencing strains with reduced levels of both cell-associated and cell-free Gp70 were generated.

### 3.2 *GP70* silencing affected *Sporothrix brasiliensis* adhesion to components of the extracellular matrix

**Fig. 3:**
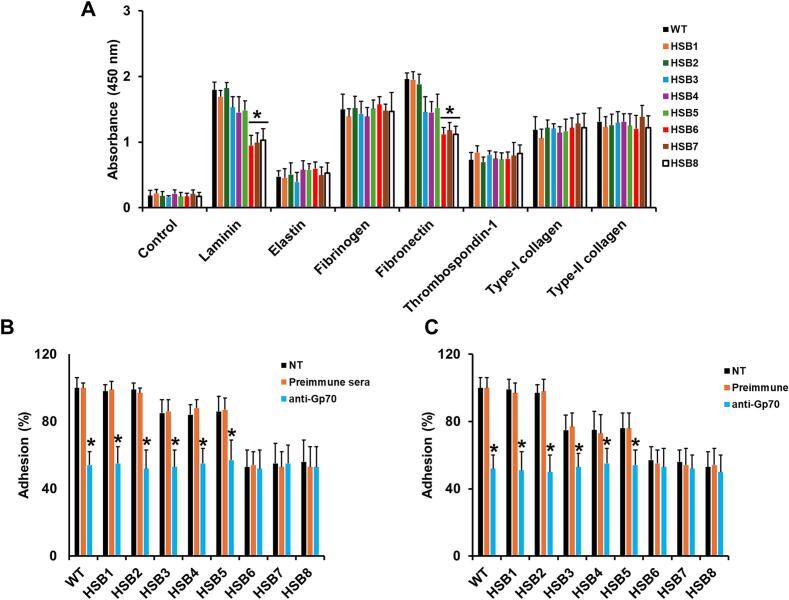
**Adhesion to extracellular matrix components of *Sporothrix brasiliensis* wild-type, control, and *GP70*-silenced strains.** In **A**, ELISA-based analysis of cell adhesion. The solid phase contained the extracellular matrix protein in 96-well plates, then yeast-like cells were added, and adherent cells were labeled with rabbit anti-rHsp60 antibodies. The presence of antibodies was detected by a colorimetric assay as described in the Material and methods section. WT, strain 5110 ATCC MYA 4823. In **B**, yeast-like cells were preincubated with preimmune sera or an anti-Gp70 antibody before their inclusion in the ELISA to detect adhesion to laminin. In **C**, the same legend as in panel **B**, but adhesion to fibronectin was analyzed. Absolute values are similar to those reported in panel **A**. Results are means ± SD of three biological replicates performed by duplicate. The Dunnett's test and then the unpaired t-test were used for data analysis. In **A**, * *P* < 0.05 when compared to WT or strains HSB1 and HSB2. In **B** and **C**, * *P* < 0.05 when compared to the non-treated (NT) condition of the same strain.

Since Gp70 was previously related to cell adhesion to extracellular matrix (ECM) components in *S. schenckii* (López-Ramírez et al., 2024), we next assessed whether *GP70* silencing had an impact on this *S. brasiliensis* trait. The WT strain showed strong adhesion to laminarin, fibrinogen, and fibronectin but moderate adhesive properties when interacting with elastin, thrombospondin-1, type-I, and type-II collagen (Fig. 3A). The control strains HSB1 and HSB2 and those with intermediate levels of *GP70* silencing (HSB3-HSB5) gave an adhesion profile similar to that observed with the WT cells (Fig. 3A). Even though the adhesion to laminin and fibronectin was reduced for HSB3-HSB5 strains, the difference was not significantly different when compared to the WT strain (*p* > 0.05 for all the cases; Fig. 3A). Strains with high *GP70* silencing levels (HSB6-HSB8) showed a significant reduction in adhesion to laminin and fibronectin; while the interaction with other ECM components was not affected (Fig. 3A). To confirm these observations, yeast-like cells were preincubated with either preimmune serum or polyclonal anti-Gp70 antibodies (Martínez-Álvarez et al., 2019) before the adhesion assays. The ability of yeast-like cells to adhere to laminin or fibronectin did not change upon preincubation with preimmune sera but adhesion to both ECM components was significantly reduced in WT, control, and HSB3-HSB5 treated with anti-Gp70 antibodies (Fig. 3B and C), confirming the role of Gp70 in the adhesion to both laminin and fibronectin. In line with this observation, strains HSB6, HSB7, and HSB8 did not show changes in the ability to bind these ECM components after interacting with anti-Gp70 antibodies (Fig. 3B and C). Therefore, Gp70 participates in the *S. brasiliensis* adhesion to laminin and fibronectin.

### 3.3 *GP70* silencing affected *Sporothrix brasiliensis* cell wall composition and organization

Since Gp70 is an abundant cell wall protein with high levels of glycosylation, we hypothesized that reduced levels may affect the cell wall composition. *S. brasiliensis* yeast-like cell walls were acid hydrolyzed and released monosaccharides were separated and quantified by HPAEC-PAD (Martínez-Álvarez et al., 2017). Following this strategy, glucosamine and glucose can be analyzed, as the monomers of the chitin and glucan polysaccharides, respectively (Martínez-Álvarez et al., 2017; García-Carnero et al., 2023). Moreover, mannose and rhamnose, the building block of glycans attached to proteins can be detected (Tamez-Castrellón et al., 2021; López-Ramírez et al., 2022).

Glucosamine was the less abundant monosaccharide within the cell wall of the WT strain, followed by glucose, rhamnose, and mannose (Table 2). The control strains and the silenced mutants HSB3-HSB5 showed a similar sugar composition to that observed in the WT strain but walls from mutant strains with high *GP70*-silencing levels showed a significant reduction in both mannose and rhamnose levels and a parallel increment in glucose content (Table 2). We also measured the cell wall protein content in the strains under analysis, and the results showed a similar protein content for the WT, control, and HSB3-HSB5 strains (see supplementary Table 1S). Even though the mutant strains with high *GP70*-silencing levels had lower protein content, this was not significantly different from the WT strain (see Table 1S; *P* = 0.0589, when compared to the protein content in the WT strain).

**Table 2:** Cell wall analysis of the *Sporothrix brasiliensis* wild-type, control, and *GP70*-silenced strains.

Cell wall abundance (μg monosaccharide mg dry cell wall^−1^)				
Organism	Glucosamine⁎	Mannose⁎	Glucose⁎	Rhamnose⁎
Wild type	40.8 ± 5.5	301.8 ± 20.6	171.7 ± 43.2	255.6 ± 20.6
HSB1	44.7 ± 6.3	298.2 ± 25.4	166.9 ± 35.4	264.9 ± 28.6
HSB2	42.0 ± 4.8	312.0 ± 29.5	176.0 ± 44.5	276.0 ± 22.4
HSB3	39.0 ± 5.9	294.5 ± 30.5	190.5 ± 45.7	266.8 ± 19.8
HSB4	43.5 ± 6.3	293.1 ± 28.7	183.3 ± 35.8	240.2 ± 24.6
HSB5	44.6 ± 4.7	291.6 ± 33.5	195.8 ± 42.8	258.1 ± 22.8
HSB6	46.8. ± 5.1	161.32 ± 38.9†	415.0 ± 42.9†	159.2 ± 26.8†
HSB7	46.7 ± 4.9	150.6 ± 41.5†	421.5 ± 35.7†	163.7 ± 32.5†
HSB8	40.4 ± 4.7	148.0 ± 37.4†	433.2 ± 39.8†	142.4 ± 21.5†

⁎ Data are means ± SD of three biological replicates.

† *P* < 0.05 when compared with the values obtained with the WT or control strains.

**Fig. 4:**
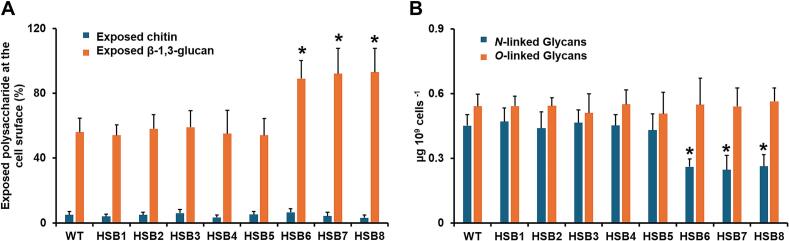
**Polysaccharide exposure at the cell wall surface and analysis of *N*-linked and *O*-linked glycans in *Sporothrix brasiliensis* wild-type, control, and *GP70*-silenced strains.** In (**A**), yeast-like cells were labeled with fluorescein isothiocyanate conjugated-wheat germ agglutinin or IgG Fc-Dectin-1 chimera for labeling of chitin and β-1,3-glucan, respectively. The fluorescence associated with 300 cells was acquired and normalized to that obtained with heat-killed cells, which represent 100 % of labeling. In (**B**), cells were treated either with endoglycosidase H or β-eliminated to trim *N*-linked glycans or *O*-linked glycans, respectively. Both were quantified by high-performance anion-exchange chromatography coupled with pulsed amperometric detection and sugar content normalized to that obtained with 1 × 10^9^ yeast-like cells. In both panels, data are means ± SD of three biological replicates performed in duplicates. The Dunnett's test and then the unpaired t-test were used for data analysis. *P < 0.05 when compared to the WT strain or control strains HSB1 or HSB2. WT, strain 5110 ATCC MYA 4823.

We also analyzed the polysaccharide distribution within the cell surface. It was previously reported that most of the chitin is hidden in the inner part of the cell wall, inaccessible to WGA-FITC, whilst a prominent amount of β-1,3-glucan is exposed at the *S. brasiliensis* cell surface (Martínez-Álvarez et al., 2017, García-Carnero et al., 2023). Here, a similar trend was observed in the WT strain, and this was replicated in the control and mutant strains with intermediate *GP70*-silencing levels (Fig. 4A). However, the mutants with high levels of *GP70* silencing (HSB6-HSB8) showed increased exposure to β-1,3-glucan at the cell surface (Fig. 4A). Chitin exposure was not affected in these mutants though (Fig. 4A). It was previously reported that changes in the cell wall components activate the cell wall integrity pathway in *S. schenckii* (López-Ramírez et al., 2022; López-Ramírez et al., 2024). Similar to other fungal species, such as *Candida albicans* and *Saccharomyces cerevisiae* (Levin, 2011; Dichtl et al., 2016), this includes Pkc1 and Mkc1 activation, which leads to the compensatory increments in structural cell wall polysaccharides (Dichtl et al., 2016). Thus the expression levels of the putative *MKC1* in *S. brasiliensis* were analyzed in the WT and mutant strains. The WT, control, and HSB3-HSB5 strains showed similar expression levels but these were upregulated 3.6 ± 0.9 times on average for strains HSB6-HSB8 (see supplementary Fig. 3S). These data indicated the activation of the cell wall integrity pathway in the latter strains.

The content of cell wall-associated *N*-linked and *O*-linked glycans was also quantified. The WT, control, and strains HSB3-HSB5 showed a similar content of *N*-linked glycans but these were significantly reduced in strains HSB6-HSB8 (Fig. 4B). No changes in the *O*-linked glycan content were observed in all the tested strains (Fig. 4B).

### 3.4 Silencing of *Sporothrix brasiliensis GP70* affected the cytokine stimulation by human peripheral blood mononuclear cells

**Fig. 5:**
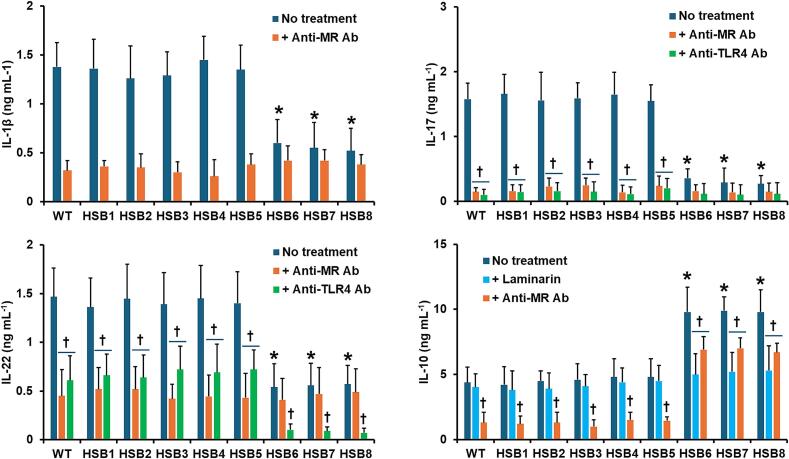
**Cytokine stimulation by human peripheral blood mononuclear cells.** Interactions between *Sporothrix brasiliensis* yeast-like cells and human peripheral blood mononuclear cells were incubated for 24 h and the secreted cytokines were recovered from supernatants and quantified by ELISA. Data are means ± SD obtained with samples from eight donors, each assayed in duplicate wells. Results were analyzed with the Dunnett's test and then Mann-Whitney *U* test. *P < 0.05 when compared to WT, HSB1, or HSB2 cells. ^†^*P* < 0.05 when compared to the “no treatment” group from the same strain. No treatment, human cells preincubated with 5 μg mL^−1^ polymyxin B; + anti-MR Ab, human cells preincubated with 5 μg mL^−1^ polymyxin B and 10 μg mL^−1^ anti-mannose receptor (MR) antibodies; + anti-TLR4 Ab, human cells preincubated with 5 μg mL^−1^ polymyxin B and 10 μg mL^−1^ anti-TLR4; + laminarin, human cells preincubated with 5 μg mL^−1^ polymyxin B and 200 μg mL^−1^ laminarin. WT, strain 5110 ATCC MYA 4823.

Next, we assessed whether changes in the cell wall composition observed in the highly *GP70*-silenced strains affected the ability of *S. brasiliensis* to stimulate both pro- and anti-inflammatory cytokines. The WT, control and silenced mutant strains stimulated similar levels of both TNFα and IL-6 (see Fig. 4S). However, the stimulation of IL-1β, IL-17, and IL-22 was reduced in the cells stimulated with the mutant strains with high levels of *GP70* silencing (HSB6-HSB8), and the opposite effect was observed for IL-10 stimulation, *i.e.*, higher cytokine levels in HSB6-HSB8-stimulated PBMCs (Fig. 5). We have previously reported that some pattern recognition receptors contribute to cytokine stimulation during the *S. brasiliensis*-PBMC interaction (Martínez-Álvarez et al., 2017, García-Carnero et al., 2023). Dectin-1 is dispensable for cytokine stimulation during this cell-cell interaction, but mannose receptor (MR), and TLR4 are the main receptors involved in the stimulation of TNFα, IL-6, IL-17, and IL-22; whilst the former participates in IL-1β and IL-10 stimulation (Martínez-Álvarez et al., 2017, García-Carnero et al., 2023). Thus, we assessed whether their contribution to cytokine stimulation was affected by *GP70* silencing. As reported, IL-1β, IL-17, IL-22, and IL-10 stimulation were dependent on MR when cells were stimulated with the WT or control yeast-like cells (Fig. 5). A similar trend was observed with the strains with intermediate silencing levels (Fig. 5). For the case of strains HSB6-HSB8, IL-1β, and IL-22 stimulation was not affected by the MR blocking, whilst this negatively affected the production of IL-17 and IL-10 (Fig. 5). The TLR4 blocking did not affect the IL-1β and IL-10 stimulation in cells interacting with WT, control, and silenced strains (see supplementary Fig. 5S). However, PBMCs preincubated with anti-TLR4 antibodies produced lower IL-17 and IL-22 levels when stimulated with the WT, control, and strains with intermediate silencing levels (Fig. 5). For the case of strains with high *GP70* silencing levels, these stimulated lower IL-17 and IL-22 levels in PBMCs preincubated with anti-TLR4 antibodies, but only the latter was statistically significant (Fig. 5). Preincubation of PMBCs with laminarin, an antagonist of dectin-1 (Rothfuchs et al., 2007; Goodridge et al., 2011) did not affect IL-1β, IL-17, or IL-22 stimulation (see supplementary Fig. 6S), nor the IL-10 stimulation with the WT, control or HSB3-HSB5 strains (Fig. 5). However, IL-10 production by laminarin-treated PMBCs stimulated with strains HSB6-HSB8 was significantly reduced. Cell-cell interaction in the presence of an irrelevant isotype-matched antibody gave cytokine levels similar to those observed in interactions with no antibody included (not shown). Collectively, these data indicate that *GP70* silencing affected the proper interaction of *S. brasiliensis* yeast-like cells and human PBMCs.

### 3.5 The *GP70*-silenced *Sporothrix brasiliensis* showed an altered interaction with human monocyte-derived macrophages

**Fig. 6:**
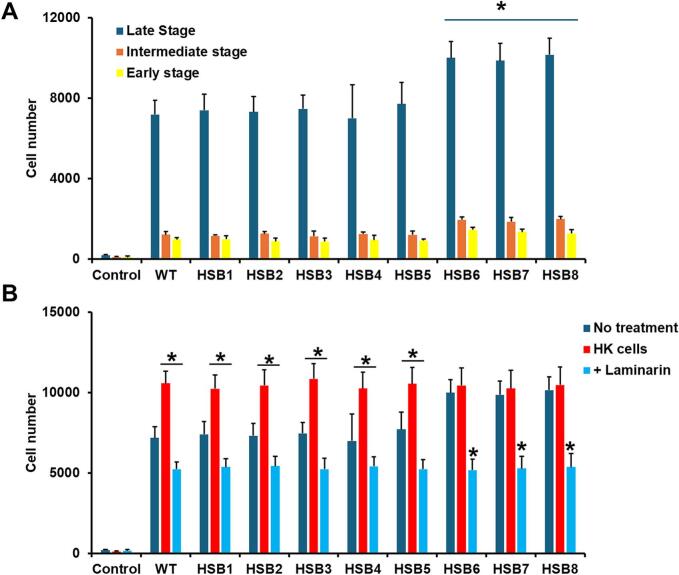
**Phagocytosis of *GP70*-silenced *S. brasiliensis* by human monocyte-derived macrophages.** In **A,** Acridine Orange-labeled yeast-like cells and human monocyte-derived macrophages were incubated for 2 h at 37 °C and 5 % (*v*/v) CO_2_, and macrophages were analyzed by flow cytometry. Fifty thousand cells were counted per sample, and only those macrophages interacting with at least one fluorescent yeast cell were included in the analysis. Depending on the positivity in different fluorescent channels, the interactions were classified as in the early, intermediate, and late stages of the uptake process. Control, mock interactions where only macrophages were included. *P < 0.05 when compared to WT, HSB1 or HSB2 strains. In **B**, the same legend as in panel **A** but yeast-like cells were either live (No treatment) or heat inactivated (HK) before the interaction with immune cells. Alternatively, the monocyte-derived macrophages were preincubated with 200 μg mL^−1^ laminarin and then challenged with live yeast-like cells (+ Laminarin). Cell numbers are from the late stage of phagocytosis. Control, macrophages interacting with no yeast-like cells. Results from both panels were analyzed with the Dunnett's test and then the Mann-Whitney U test. *P < 0.05 when compared to the “no treatment” condition of the same strain. In both panels, results are means ± SD from eight donors assayed by duplicate. WT, strain 5110 ATCC MYA 4823.

Next, we analyzed whether *S. brasiliensis* phagocytosis was affected by the *GP70* silencing. For this purpose, we differentiated peripheral blood monocytes into macrophages (Perez-Garcia et al., 2016). The fungal uptake was analyzed by flow cytometry, and results showed that most monocyte-derived macrophages were in the late stage of phagocytosis when interacting with WT, control, or mutant strains with intermediate *GP70*-silencing levels, followed by cells in the intermediate and early stages (Fig. 6A). Immune cells interacting with strains HSB6-HSB8 showed increased ability to phagocyte *S. brasiliensis* and higher cell numbers were observed in the late, intermediate, and early stages of phagocytosis (Fig. 6A).

A similar phagocytic profile has been previously reported in *S. schenckii* silenced mutants in *ROT2* and *RmlD*, and this was associated with increased β-1,3-glucan levels at the cell wall surface (Tamez-Castrellón et al., 2021; López-Ramírez et al., 2022). So, we tested whether a similar hypothesis may apply here. The heat-killed (HK) cells artifactually expose inner wall components at the cell surface, and consequently, the structural polysaccharides are more available to interact with receptors than live cells (Gantner et al., 2005; Gow et al., 2007). Here, monocyte-derived macrophages showed increased levels of uptake when interacting with HK yeast-like cells from the WT, control, and HSB3-HSB5 strains (Fig. 6B) These were to the same extent as those cell numbers in the interactions with mutants HSB6-HSB8, which did not show a significant increment when HK cells were used in the interactions (Fig. 6). When the phagocytosis analysis was performed with immune cells preincubated with laminarin, the uptake levels were significantly reduced in macrophages incubated with WT, control, or silenced strains (Fig. 6B), indicating a significant role of dectin-1 during the *S. brasiliensis*-monocyte-derived macrophage interaction. These results showed an abnormal phagocytosis of strains with high levels of *GP70* silencing.

### 3.6 Interaction of *Sporothrix brasiliensis GP70*-silenced mutant strains with *Galleria mellonella* larvae

**Fig. 7:**
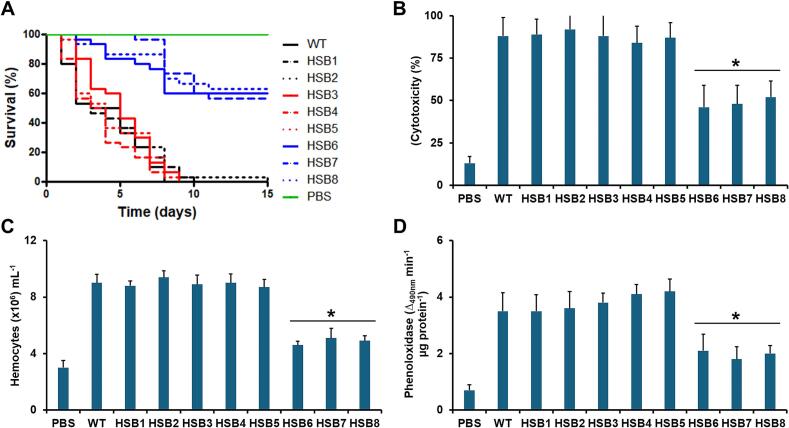
**Analysis of virulence in *Galleria mellonella* larvae.** In **A,** Groups containing 30 larvae were inoculated with 1 × 10^5^ yeast-like cells and survival followed for two weeks. Data are shown in Kaplan–Meier plots. The Log-rank test showed no differences among the WT and HSB1-HSB5 strains (*P* = 0.87). Mortality curves generated with strains HSB6-HSB8 were significantly different from those generated with WT cells (*P* < 0.05). In **B**. hemolymph was collected and used to measure cell-free lactate dehydrogenase. The 100 % value corresponds to that obtained from lysed hemocytes. This hemolymph was also used to quantify hemocytes (**C**) or phenoloxidase activity (**D**). PBS refers to an animal group inoculated with phosphate saline buffer. WT, strain 5110 ATCC MYA 4823. In **A**, each group contained 30 larvae, In **B**, **C**, and **D**, data are shown as means ± SD from three biological replicates. Each animal group contained 10 larvae, and results were analyzed with the Dunnett's test and then the Mann-Whitney U test. *P < 0.05 when compared to the animal group inoculated with the WT, HSB1, or HSB2 control strains.

Finally, to assess the contribution of *GP70* to *S. brasiliensis* virulence, we injected yeast-like cells into the hemocele of *G. mellonella* larvae and measured animal mortality, cytotoxicity, and cellular and humoral immunity effectors. This model was chosen because generates mortality profiles similar to those observed in mice and is useful for assessing immunological priming, an immunological immune response in insects similar to the adaptive immunity observed in mammalian hosts (Clavijo-Giraldo et al., 2016, Lozoya-Pérez et al., 2020, Macêdo-Sales et al., 2020, Sheehan et al., 2020, Reis and de Jesus, 2023). Animals inoculated with the WT or HSB1-HSB2 control strains successfully established a systemic infection and killed 100 % of the animal population, with a median survival of 4.0 ± 1.0 days (Fig. 7A). Larvae groups inoculated with the strains with intermediate levels of *GP70* silencing (HSB3-HSB5) showed mortality curves similar to those generated with either WT or control strains (Fig. 7A). Contrary to these groups, those inoculated with the strains HSB6-HSB8 showed similar mortality curves where 59.5 ± 4.0 % of animals survived during the two-week observation, with a median survival of >15 days (Fig. 7A). The two animal groups were significantly different from those inoculated with the WT or control strains (*P* < 0.05 in all cases). Fungal colony-forming units in hemolymph were similar for all the animal groups (see supplementary Table 2S), suggesting all three strains showed similar adaptation to the host milieu. When highly *GP70*-silenced fungal cells were collected from the hemolymph of both dead and alive larvae these were forming small cell aggregates or did not form aggregates at all. This observation suggests that cell aggregation may not occur in the *in vivo* setting. Even though we cannot rule out the possibility that during early post-inoculation times cells were aggregating, the observation suggests that cell aggregation may not affect the virulence results. Cytotoxicity, hemocyte levels in the hemolymph, and phenoloxidase activity showed low levels in the animal groups inoculated with the highly *GP70*-silenced mutant strains (HSB6-HSB8) when compared to the WT, control, and mutant strains with intermediate *GP70* silencing levels (Fig. 7B, C, and D). Collectively, these data suggest that virulence is affected by *GP70* silencing in *S. brasiliensis*.

## 4 Discussion

*S. brasiliensis* causes sporotrichosis, an acute or chronic granulomatous subcutaneous mycosis that affects human beings and other mammals (Mora-Montes et al., 2015). Similar to other medically relevant fungal species, the cell wall modulates the interaction with the host cells and tissues and contains species-specific components (Netea et al., 2008, Arana et al., 2009, Díaz-Jiménez et al., 2012, Martinez-Alvarez et al., 2014, Martínez-Álvarez et al., 2017, Lopes-Bezerra et al., 2018a, Garcia-Rubio et al., 2020). Thus far, the contribution of particular genes to *S. brasiliensis* pathogenicity and virulence is limited, and currently, only the CRISPR-Cas9 methodology has been applied to study the role of *S. brasiliensis* melanin in virulence (Hatinguais et al., 2023). Here, *GP70* silencing at high levels led to cell aggregation and smaller yeast-like cells, an observation that contrasts with the *S. schenckii GP70*-silenced strains (López-Ramírez et al., 2024). We showed this was related to an abnormal β-glucan content at the cell wall, as treatment with glucanase solved cell aggregates. Currently, the analysis of *S brasiliensis* glucans is limited, and there are only reports about the presence of β-1,3-glucan (Martínez-Álvarez et al., 2017; Villalobos-Duno et al., 2021; García-Carnero et al., 2023). However, *S. schenckii* glucans are diverse, as soluble glucans have proportions of 44 %, 28 %, and 28 % for β-1,3-, β-1,6-, and β-1,4-glucan, respectively; and in the case of insoluble glucans, the proportions are 66 %, 29 %, and 5 % for β-1,3-, β-1,6-, and β-1,4-glucan, respectively (Previato et al., 1979). Assuming these other glucans and proportions are also found in *S. brasiliensis*, it is possible to speculate cell aggregates are linked to the increased content in β-1,3-glucan. However, the glucanase used here has β-1,3- and β-1,4-endoglucanase activities (Bauer et al., 1973; Kanda et al., 1976). Consequently, this cell phenotype may also be related to abnormal levels of β-1,4-glucan, if present. Another possible explanation for this observation may be linked to the decreased levels in the *N*-linked glycan content when *GP70* is highly silenced. *Candida albicans* and *Saccharomyces cerevisiae* with defects in the *N*-linked glycosylation pathway have shown clumpy phenotypes, where aggregates are solved by incubating cells with chitinases or glucanases (Nagasu et al., 1992; Bickle et al., 1998; Bates et al., 2005; Bates et al., 2006; Mora-Montes et al., 2007). It has been hypothesized that cell aggregation in these organisms is a consequence of the increment in structural cell wall polysaccharides, which delay cell detachment at the end of the budding process (Bates et al., 2005; Bates et al., 2006). Nevertheless, it is important to stress that *S. schenckii* silenced strains with increased levels of cell wall β-glucan content (Tamez-Castrellón et al., 2021; López-Ramírez et al., 2022; López-Ramírez et al., 2024) did not show this kind of phenotype, suggesting that β-glucans content, type, and distribution within the cell wall are different between *S. schenckii* and *S. brasiliensis*. This plays a relevant role in *Sporothrix* pathogenicity, as the cell wall β-glucan content has been related to *Sporothrix* virulence (Villalobos-Duno et al., 2021). It is worth mentioning that our cell wall analysis did not include the analysis of cell wall glycolipids (Carlos et al., 2003; Rodrigues et al., 2008; Carlos et al., 2009), which represents a limitation of our cell wall characterization.

The 3-carboxy-cis,cis-muconate cyclase activity was expected to be present in *S. brasiliensis*, as predicted by the amino acid sequence similarity between the Gp70 from this species and *S. schenckii* (Rodrigues et al., 2015). The fact that a significant amount of this enzyme was found in cell-free extracts and the use of yeast-like cells undergoing dimorphism for four days and not ten days, may explain the discrepancy between these results and those previously reported, where a bare detection of Gp70 was observed in *S. brasiliensis* cell wall (Castro et al., 2013). The Gp70 bioinformatics analysis predicts a canonical signal peptide, with a cleavage site between positions 21 and 22 [analyzed with SignalP-6.0 (Teufel et al., 2022)], suggesting the glycoprotein is transported to the extracellular compartment *via* a canonical secretory pathway. This evidence, along with its immunolocalization in the cell wall (Castro et al., 2013) and the promising results of immunotherapy approaches targeting *S. brasiliensis* Gp70 (de Almeida et al., 2015; de Almeida et al., 2017; de Almeida et al., 2018) suggest that Gp70 is a key component of the cell wall. Currently, it is not known the contribution of 3-carboxy-cis,cis-muconate cyclase activity to *Sporothrix* biology, and the working model for this enzyme places it in the benzoate degradation pathway, a metabolic route likely to be active when growing on vegetable matter (Rodrigues et al., 2015). However, soluble Gp70 may have a role in immunoevasion, disguising humoral and cellular immune effectors, as reported for other fungal pathogens (Hernández-Chávez et al., 2017). It is worth noting that Gp70 has not been detected within the *S. brasiliensis* extracellular vesicles (Ikeda et al., 2018). Because of the Gp70 enzyme activity and the adhesive properties, this is likely a moonlighting protein.

*S. brasiliensis* adhesion to host components is currently a subject barely studied, and thus far, only the adhesion to plastic surfaces during biofilm formation has been explored in this fungal species (Garcia et al., 2020). The *S. brasiliensis* yeast-like cells showed a similar adhesion profile to that observed with *S. schenckii* yeast-like cells (García-Carnero et al., 2021; López-Ramírez et al., 2024), with exception of thrombospondin-1, which is not a ligand for *S. schenckii* adhesins (Lima et al., 1999; García-Carnero et al., 2021; López-Ramírez et al., 2024). Since thrombospondin-1 is involved in the regulation of cellular components of both innate and adaptive immunity (Kaur and Roberts, 2024), we can speculate that adhesion to this ECM may affect its regulatory functions, giving *S. brasiliensis* an advantage to colonize and affect host tissues. This may contribute to explaining the higher virulence associated with *S. brasiliensis*. The *S. schenckii* Gp70 was identified as an adhesin to laminin and fibronectin, a result that was replicated here. This is likely to be explained by the already mentioned high similarity between both proteins.

The cell wall changes in the *S. brasiliensis* strains with high *GP70* silencing levels were similar to that observed in *S. schenckii GP70* silenced strains (López-Ramírez et al., 2024), suggesting that Gp70 has a similar role in both species. The cell wall integrity pathway responded in the same way in both species and the compensatory mechanism involved only increased β-1,3-glucan levels exposed at the cell wall surface (López-Ramírez et al., 2024). The low mannose, rhamnose, and cell wall protein contents indicate this is an abundant and highly glycosylated cell wall protein, as previously reported (Rodrigues et al., 2015; Martínez-Álvarez et al., 2019).

The cytokine stimulation by human PBMCs showed a strong dependence of cytokine production on MR, an observation that contrasts with the interaction between human PBMCs and *S.schenckii*, where cytokine stimulation is linked to activation of dectin-1, TLR2, and TLR4 (Martínez-Álvarez et al., 2017; García-Carnero et al., 2023). These results are in line with previous observations indicating that cell wall components between both species are different and therefore stimulate differentiated cytokine profiles (Kischkel et al., 2022). Interestingly, TLR4 was not the dominant pattern-recognition receptor when interacting with *GP70*-silenced strains, as reported in *S. schenckii* (López-Ramírez et al., 2024), stressing the fact that the cell wall composition of both species is different (Lozoya-Pérez et al., 2020; Villalobos-Duno et al., 2021; García-Carnero et al., 2023). When interacting with monocyte-derived macrophages, dectin-1 was the main player during the *S. brasiliensis*-macrophage interaction, which contrasts with *S. schenckii* cells.

Similar to *S. schenckii*, *S. brasiliensis GP70* silencing led to virulence attenuation in the *G. mellonella* model (López-Ramírez et al., 2024). This may imply that Gp70 is a key adhesin for virulence also in *S. brasiliensis*, supporting the observations that monoclonal antibodies against this glycoprotein are enough to protect animals from sporotrichosis (de Almeida et al., 2015; de Almeida et al., 2017). A similar observation has been reported for *Sporothrix globosa* (Chen et al., 2017). The alternative explanation for these results is that rhamnose levels are reduced in the cell wall of mutants with high *GP70* silencing levels, and reduction of this cell wall component has been linked to decreased virulence (Tamez-Castrellón et al., 2021; Villalobos-Duno et al., 2021). However, this may be unlikely, as the remaining rhamnose levels in the cell walls of these cells, even though reduced, represent about 25 % of the total cell wall sugar content.

Our results indicate that highly *GP70*-silenced mutant strains induced lower levels of immune effectors than the WT strain. This may suggest that immune functions, such as phagocytosis, are not positively affected. This discrepancy with the results obtained with human cells may rely on the nature and abundance of receptors involved in the phagocytic process in both human and insect cells (Lavine and Strand, 2002).

In conclusion, *S.brasiliensis GP70* encodes for a 3-carboxy-cis,cis-muconate cyclase with properties of adhesin to laminin and fibronectin. This is an abundant cell wall glycoprotein that contributes to the interaction with cells of innate immunity and also in fungal virulence.

The following are the supplementary data related to this article.

**Supplementary Fig. S2:**
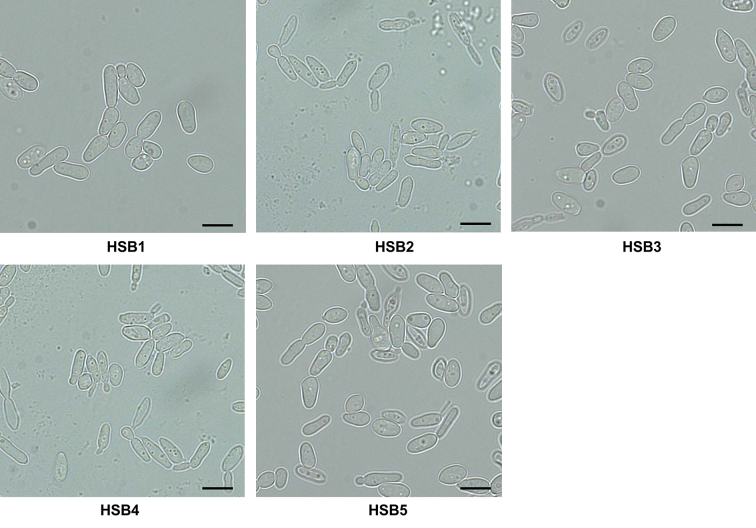
***Sporothrix brasiliensis* yeast-like cell morphology*.*** Representative light microscopy images of *S. brasiliensis* control cells (HSB1 and HSB2) and three mutant strains with intermediate levels of *GP70* silencing (HSB3, HSB4, and HSB5).Scale bars = 5.0 μm.

**Supplementary Fig. S3:**
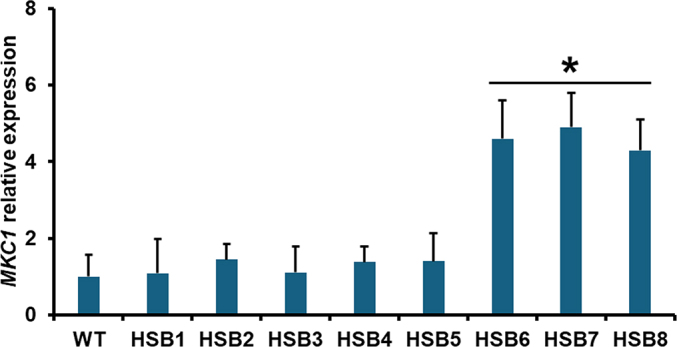
**Analysis of *MKC1* expression in *Sporothrix brasiliensis* wild-type, control, and *GP70*-silenced strains.** Four-days-grown yeast-like cells were harvested, total RNA was isolated and *MKC1* expression was analyzed by RT-qPCR, as described in Materials and methods. The expression of the gene encoding the ribosomal protein L6 was used for data normalization. Results are means ± SD of three biological replicates. Data were analyzed with Dunnett's test and then the unpaired t-test. * *P*< 0.05 when compared to WT, strain HSB1 or HSB2. WT, strain 5110 ATCC MYA 4823.

**Supplementary Fig. S4:**
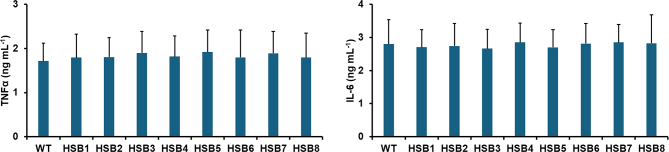
**TNFα and IL-6 stimulation by human peripheral blood mononuclear cells.** Interactions between *Sporothrix brasiliensis* yeast-like cells and human peripheral blood mononuclear cells were incubated for 24 h and the secreted TNFα and IL-6 were quantified by ELISA. Data are means ± SD obtained with samples from eight donors, each assayed in duplicate wells. Data were analyzed with Dunnett's test, *P* = 0.5687. WT, strain 5110 ATCC MYA 4823.

**Supplementary Fig. S5:**
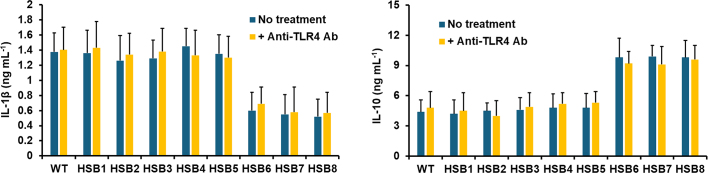
**IL-1β and IL-10 stimulation by human peripheral blood mononuclear cells.** Interactions between *Sporothrix brasiliensis* yeast-like cells and human peripheral blood mononuclear cells were incubated for 24 h and the secreted IL-1β and IL-10 were quantified by ELISA. Data are means ± SD obtained with samples from eight donors, each assayed in duplicate wells. Data were analyzed with Dunnett's test, *P* = 0.1567. No treatment, human cells preincubated with 5 μg mL^-1^ polymyxin B; + anti-TLR4 Ab, human cells preincubated with 5 μg mL^-1^ polymyxin B and 10 μg mL^-1^ anti-TLR4. WT, strain 5110 ATCC MYA 4823.

**Supplementary Fig. S6:**
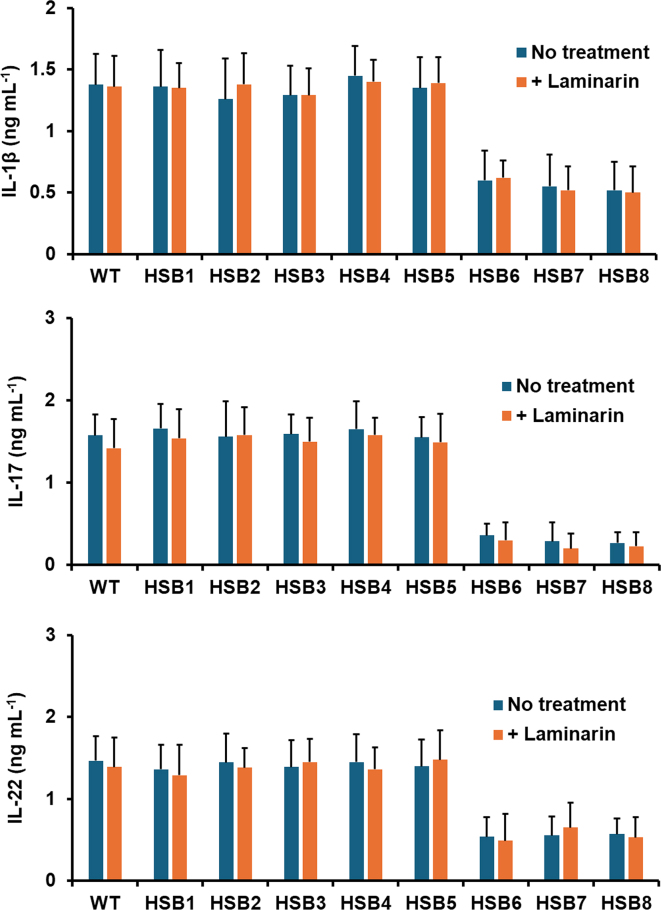
**IL-1β, IL-17, and IL-22 stimulation by human peripheral blood mononuclear cells.** Interactions between *Sporothrix brasiliensis* yeast-like cells and human peripheral blood mononuclear cells were incubated for 24 h and the secreted cytokines were quantified by ELISA. Data are means ± SD obtained with samples from eight donors, each assayed in duplicate wells. Data were analyzed with Dunnett's test, *P* = 0.2466. No treatment, human cells preincubated with 5 μg mL^-1^ polymyxin B; + laminarin, human cells preincubated with 5 μg mL^-1^ polymyxin B and 200 μg mL^-1^ laminarin. WT, strain 5110 ATCC MYA 4823.

Supplementary material 1 Supplementary material 1

Supplementary material 2 Supplementary material 2

## CRediT authorship contribution statement

**Leonardo Padró-Villegas:** Writing – review & editing, Visualization, Methodology, Investigation, Formal analysis, Data curation, Conceptualization. **Manuela Gómez-Gaviria:** Writing – review & editing, Validation, Methodology, Investigation, Formal analysis. **Iván Martínez-Duncker:** Writing – review & editing, Validation, Supervision, Resources, Methodology, Investigation, Formal analysis, Data curation, Conceptualization. **Luz A. López-Ramírez:** Writing – review & editing, Supervision, Project administration, Methodology, Investigation, Formal analysis. **José A. Martínez-Álvarez:** Writing – review & editing, Validation, Methodology, Investigation, Formal analysis. **Gustavo A. Niño-Vega:** Writing – review & editing, Supervision, Resources, Methodology, Investigation, Formal analysis, Data curation, Conceptualization. **Héctor M. Mora-Montes:** Writing – review & editing, Writing – original draft, Validation, Supervision, Resources, Project administration, Methodology, Funding acquisition, Formal analysis, Data curation, Conceptualization.

## Declaration of competing interest

The authors declare that they have no known competing financial interests or personal relationships that could have appeared to influence the work reported in this paper.
