# Supplementary material for: Sporothrix brasiliensis Gp70 is a cell wall protein required for adhesion, proper interaction with innate immune cells, and virulence
Source: Cell Surf. 2025 Jan 6;13:100139. doi: 10.1016/j.tcsw.2024.100139 (PMC11763198; doi:10.1016/j.tcsw.2024.100139)
Supplement: Supplementary material 1 [file mmc7.docx]

**Table 1S. Cell wall protein content of the *Sporothrix brasiliensis* wild-type, control, and *GP70*-silenced strains**

| Organism | Protein* |
| --- | --- |
| Wild type | 198.8 ± 40.6 |
| HSB1 | 188.6 ± 36.4 |
| HSB2 | 180.4 ± 22.5 |
| HSB3 | 182.7 ± 39.8 |
| HSB4 | 184.2 ± 32.6 |
| HSB5 | 189.8 ± 23.5 |
| HSB6 | 158.9 ± 38.5 |
| HSB7 | 155.2 ± 41.5 |
| HSB8 | 149.8 ± 37.7 |

* µg mg dry cell wall^-1^. Data are means ± SD of three biological replicates.
