# Supplementary material for: Sporothrix brasiliensis Gp70 is a cell wall protein required for adhesion, proper interaction with innate immune cells, and virulence
Source: Cell Surf. 2025 Jan 6;13:100139. doi: 10.1016/j.tcsw.2024.100139 (PMC11763198; doi:10.1016/j.tcsw.2024.100139)
Supplement: Supplementary material 2 [file mmc8.docx]

**Table 2S. Fungal burden in *Galleria mellonella* inoculated with wild-type, control, and *GP70*-silenced *Sporothrix brasiliensis*.**

| **Strain** | **Colony-Forming Units (×10^5^) ^a^** |
| --- | --- |
| PBS ^b^ | 0.0 ± 0.0 |
| WT ^c^ | 2.5 ± 0.6 |
| HSB1 | 2.6 ± 0.5 |
| HSB2 | 2.9 ± 0.7 |
| HSB3 | 2.6 ± 0.4 |
| HSB4 | 2.5 ± 0.7 |
| HSB5 | 2.5 ± 0.6 |
| HSB6 | 2.8 ± 0.5 |
| HSB7 | 2.9 ± 0.8 |
| HSB8 | 2.8 ± 0.9 |

^a^ Surviving and dead animals were decapitated and the hemolymph was collected and used to calculate the colony-forming units by incubating on YPD plates. Data are means ± SD of hemolymph collected from 30 larvae per group.

^b^ Control group inoculated only with PBS.

^c^ WT, strain 5110 ATCC MYA 4823. Strains HSB1 and HSB2 were transformed with pBGgHg; while HSB3-HSB8 with pBGgHg-GP70.
